# Substitution or Dilution? Assessing Pre-Fermentative Water Implementation to Produce Lower Alcohol Shiraz Wines

**DOI:** 10.3390/molecules25092245

**Published:** 2020-05-10

**Authors:** Olaf J. Schelezki, Alain Deloire, David W. Jeffery

**Affiliations:** 1 Department of Wine and Food Science, and Waite Research Institute, The University of Adelaide (UA), Waite Campus, PMB 1, Glen Osmond, SA 5064, Australia; Olaf.Schelezki@lincoln.ac.nz; 2 Australian Research Council Training Centre for Innovative Wine Production, UA, Waite Campus, PMB 1, Glen Osmond, SA 5064, Australia; 3 Department of Biology-Ecology, University of Montpellier, L’institut Agro (SupAgro), 2 Place Pierre Viala, 34060 Montpellier, France; alain.deloire@supagro.fr

**Keywords:** wine quality, polyphenols, tannins, wine colour, sensory, descriptive analysis, climate, harvest timing

## Abstract

Changes to regulations by Food Standards Australia New Zealand have permitted the adjustment of must sugar levels with the addition of water in order to ensure a sound fermentation progress as well as mitigating excessive wine–alcohol levels. This study assessed the implications for Shiraz wine quality following a pre-fermentative must dilution (changing liquid-to-solid ratios), in comparison to juice substitution with water (constant liquid-to-solid ratios) that has previously been deemed a promising way to adjust wine–alcohol levels. While working within the legal limit of water addition to grape must, the effects of both approaches on wine quality parameters and sensory characteristics were rather similar, and of negligible nature. However, different implications between substitution and dilution appeared to be driven by grape maturity, and dilution was found to have a greater impact than substitution on some parameters at higher water implementation rates. In line with previous observations, longer hang-time followed by alcohol adjustments via pre-fermentation water addition were of limited merit compared to simply picking grapes earlier. This work provided further knowledge that supports informed decision making regarding the recently permitted approach of using water during winemaking.

## 1. Introduction

Pre-fermentative winemaking interventions have drawn attention in recent years to provide options that help mitigate elevated alcohol levels in red wines resulting from climate change related shifts in grapevine phenology and the trend toward increased grape total soluble solids (TSS) levels at harvest [1,2,3,4,5]. Specifically, after removing certain amounts of juice (saignée) from a red grape must, equal proportions of a very low alcohol wine (green harvest wine, GHW), resulting from fruit harvested at véraison (approximately 8 °Brix) or water were added to dilute must TSS concentrations and yield wines with decreased alcohol levels. Those studies variously assessed the implications on wine colour, tannin and volatile compositions as well as the effects on wine sensory profiles. In the case of Cabernet Sauvignon, pre-fermentative substitution did not have any adverse effects on parameters such as anthocyanin concentration, colour intensity, the proportion of stable pigments, or tannin concentration and molecular mass (MM), across different vintage conditions [1,3,5]. In one case, lower alcohol Cabernet Sauvignon wines resulting from water implementation were superior in colour intensity, stable pigment formation and tannin concentration compared to the control [5].

Volatile profiles and sensory characteristics of the lower alcohol Cabernet Sauvignon wines seemed to be reflective of the traits determined by the fruit harvested at commercial maturity when using pre-fermentative substitution with water, but differences according to the grape maturity were apparent [2,5]. That is, in contrast to a season with evident berry shrivel where over-ripe sensory characters were maintained in the water treatment wines, a “normal” grape maturity context (milder ripening season) showed a general decline in concentration and higher proportion of affected volatile compounds with higher levels of water implementation [5]. Nonetheless, other than potentially harvesting grapes earlier, water was identified as being the more favourable choice in comparison to producing a GHW to manage wine–alcohol levels, especially given the lower costs and simplicity afforded by using water. More research is required, however, in light of previous findings indicating that the apparently benign observations mentioned above do not necessarily apply with cultivars other than Cabernet Sauvignon. For instance, Sherman et al. [6] and Schelezki, Antalick, Šuklje and Jeffery [5] demonstrated decreasing tannin concentrations with higher water substitution rates (and more so with GHW [5]) in Merlot and Shiraz wines, respectively, in combination with negative impacts on colour density and stability in the Shiraz wines, which remained evident after 12 months of bottle ageing [5].

As the most widely grown cultivar in Australia [7], Shiraz is of significant value to the wine industry, but it is also a variety that is particularly susceptible to berry shrivel [8], which results in higher berry sugar concentrations and increased wine–alcohol levels. This implies a prospective role of pre-fermentative alcohol management for Shiraz in Australia (and likely elsewhere) and studies that build on the previous findings are required, such as the one by Teng et al. [9]. Furthermore, according to the Food Standards Australia New Zealand, water can be added to grape must before commencing fermentation to yield a minimum of 13.5 °Baumé (Bé) without the need of removing juice [10]. Thus, in contrast to the previous approach of proportional substitution of juice with water so as not to alter the solid–liquid ratio [5], the effectively higher wine volumes and easier implementation of adding water without removing juice could be more favourable for wine producers. However, the consequences for wine quality of changing the solid–liquid ratio by diluting the must with water remained to be investigated.

This study therefore aimed to (i) provide a direct comparison between the two variants of pre-fermentative water addition (substitution, S, versus dilution, D) to manage wine–alcohol levels through the evaluation of wine colour and tannin parameters, and wine sensory attributes of lower alcohol Shiraz wines, and (ii) assess the implications for wine quality as a result of grape maturity by harvesting Shiraz grapes at two distinct maturity levels, termed fresh fruit (FF) and mature fruit (MF), for the implementation of pre-fermentative water addition regimes.

## 2. Results and Discussion

### 2.1. Vintage Conditions and Fruit Parameters

With 2323 Huglin index units, the 2016/2017 growing season of McLaren Vale was classified as temperate warm [11], and was therefore significantly cooler than the preceding vintage 2015/2016 [5]. Lower than average temperatures from September to December 2016 in conjunction with above average monthly rainfall from the beginning of the season (except for a drier November, Table 1) caused a lagging phenological development that delayed the commercial harvest date by almost a month compared to the previous year [5].

The average fresh weight of a population of berries remained constant between FF to MF harvests (Table 2), indicating the absence of vine water constraint until commercial harvest [12], whereas TSS levels increased from 22.7 °Brix in FF to 25.5 °Brix in MF. The grape TSS increments were in line with rising pH and decreasing TA concentrations. Grapes from the later harvest point were further characterised by higher extractable tannin (expressed in mg/g berry, Table 2), similarly to what was observed previously for the same vineyard under warmer and drier vintage conditions [5]. The higher amount of extractable tannin per berry in the present study compared to the previous one was deemed likely to result from pre-véraison accumulation within the berry [8] rather than a concentration effect [1].

### 2.2. Basic Wine Composition

All wines were fermented to dryness (<1 g/L of total sugar, Table 3). The FF_Control and MF_Control wines yielded 13.6% and 15.5% alcohol by volume (ABV), respectively. For the FF harvest, water implementation rates of 41.0%, 26.6% and 11.6% v/v resulted in lower alcohol wines with 9.6%/9.0% ABV in FF_S1/D1, 11.1%/10.8% ABV in FF_S2/D2 and 12.6%/12.6% ABV in FF_S3/D3, respectively (Table 3 and Figure S1 of the Supporting Information). With the MF fruit, implementation rates of 47.2%, 34.0% and 10.2% v/v of water produced lower alcohol wines with 10.6%/9.6%, 12.0%/11.7% and 14.5%/14.4% ABV, respectively. Acetic acid concentration tended to remain constant across the wines, except for lower values in FF_S3/D3 compared to the control. A decline in malic acid concentration was noted; however, it only became significant in FF_D1 and MF_D1/S1 wines. Furthermore, while the pH within each of the substitution treatments (FF_S1–S3 and MF_S1–S3) were of similar levels, using identical addition rates of tartaric acid solution to adjust the dilution treatment wines resulted in significant declines in pH, likely due to a decreased buffer capacity [13]. Acetaldehyde and glycerol levels decreased with lower established alcohol levels and to similar extents regardless of whether substitution or dilution was employed (Table 3). As glycerol forms part of the dry extract of wines, the lower levels resulting from the water implementations could translate into differing mouthfeel properties, such as viscosity or astringency [14].

### 2.3. Colour and Tannin Properties

The perceived quality and value of red wines critically relates to the sensory attributes, some of which result from wine colour and tannin properties [15]. Later harvest dates have been associated with higher colour intensities and tannin concentrations [5,16,17], but longer grape hang-times may be associated with additional vineyard costs (i.e., irrigation) or added risk of berry shrivel. Ideally, the implications on colour and tannin parameters following pre-fermentative water addition to manage alcohol levels should be minimal to retain the wine style as determined by harvest date, so that taking the risk of later harvest to target a particular style of wine remains justifiable.

With a later harvest date (FF_Control versus MF_Control wines), there were increases in wine colour intensity, total anthocyanins, total phenolics, SO_2_-resistant pigments, and tannin concentration and MM (Table 4), which was consistent with previous observations for the same vineyard [5]. Notably, despite being of comparable alcohol levels resulting from analogous grape TSS values at harvest, anthocyanin concentrations in the MF_Control (944 mg/L) exceeded the level at commercial harvest reported in the study for the preceding vintage (590 mg/L), which was characterised by significantly warmer growing conditions [5]. A study by Sadras and Moran [18] explained that anthocyanin–TSS ratios are likely to be disrupted by elevated growing season temperatures, which delay the onset of anthocyanin accumulation in the berry skin. On the other hand, lower wine tannin concentration was determined in the MF_Control wine (917 mg/L) in comparison to the preceding vintage (1256 mg/L) [5], albeit of comparable MM values (1640 g/mol).

The lower tannin concentration could be attributable to the lower growing season temperature, as was similarly observed previously [19]. Nonetheless, the wine tannin concentration increment from FF_Control to MF_Control (Table 4) was reflective of the increased grape extractable tannins determined with the wine-like extraction protocol (Table 2), which was also the case previously (although less obvious) [5]. However, the observed difference in tannin concentration between the commercial harvest (i.e., last harvest date) wines of the present and previous vintages (that had similar TSS and ABV levels) was not represented by the wine-like assay. This might be indicative of differences in tannin extraction dynamics during winemaking as a function of the vintage conditions and grape ripening phenomena, which was not mirrored by the wine-like extracts.

In particular, it was previously shown that grape skin cell walls become more porous with longer grape hang-times [20], which increases their affinity to bind tannins and remove them from the wine solution [21]. Furthermore, differences in seed tannin extractability between these vintages may have been present [22], despite the similar TSS concentrations, given that harvest was significantly delayed in the present study compared to the previous one [5], indicating the decoupling between primary and secondary grape metabolites. Thus, an enhanced seed tannin extractability may have resulted in final higher tannin concentrations in the 2016 commercial harvest.

Substituting 11.6% v/v of FF juice with water (FF_S3), affording a decrease of 1% ABV in the wine compared to FF_Control, did not significantly affect wine colour parameters and phenolics measures (Table 4). However, adding the same proportion of water without conducting saignée (i.e., juice run-off) provoked a lower level of total anthocyanins, total phenolics and stable pigments in the respective wine (FF_D3), albeit without an impact on wine colour density (Table 4). Overall, dilution had a more pronounced effect compared to substitution, in contrast to previous results [9], thereby highlighting the importance of the method of water incorporation to manage wine–alcohol levels.

The divergence between the modes of water implementation becomes increasingly evident in wines with lower established alcohol levels. The dilution treatments (FF_D1 and D2) resulted in inferior colour parameters than in the respective substitution treatment (FF_S2 and S1) and FF Control wines. At a dilution rate of 41.0% v/v, colour intensity, anthocyanin concentration, SO_2_-resistant pigments and total phenolics decreased in the order of 32%–35%, whereas substitution with the equivalent volumes of water lowered these colour and phenolic parameters by only 11%–18% (Table 4). Although less pronounced, tannin concentrations followed a similar trend, but a divergence only became significant in the lowest alcohol wine (i.e., FF_D1/S1). Must dilution with 41.0% v/v of water in FF_D1 resulted in 61% less tannin compared to the control, whereas the juice substitution counterpart FF_S1 did not significantly differ from the control. Despite not being different to FF_S1 or FF_D2, a significantly lower tannin concentration was found for FF_S2 compared to the control.

The tannin results may indicate that at a dilution level of up to about 26% v/v as applied in FF_D2, enough tannin was extractable and retained in the wine to establish an equilibrium similar to the FF_S2 treatment that had a constant solid–liquid ratio [23]. Additionally, tannin MM results appeared to be more sensitive to the dilution treatments, as a lower MM was already noticeable by FF_D2 (11% ABV) compared to the control, whereas substituting juice with the equivalent amount of water retained the tannin MM as defined by the FF harvest date (i.e., as in FF_Control). However, at the highest substitution rate in FF_S1, the tannin MM was significantly lower in relation to FF_Control, and similar to the FF_D2 treatment (which also had the same tannin concentration, Table 4). It could be possible that a proportion of the observed decline in tannin concentration with water implementation was due to the loss of higher MM tannin, given a higher binding affinity with wine matrix constituents (i.e., proteins or polysaccharides) or grape cell walls at lower alcohol concentrations [24,25]. Ultimately, however, the cause or relevance to wine chemical and sensory properties was unresolved.

The more pronounced effect on wine colour and phenolic characteristics caused by diluting the must rather than substituting a proportion of juice was also evident among the MF wines (Table 4). Interestingly, among the water implementation treatments for the MF harvest, lower values for colour density, anthocyanins, phenolics and SO_2_-resistant pigments were already evident (Table 4) in wines that were lower in alcohol by only 1% ABV, resulting from either dilution or substitution with 10.2% v/v of water (Table 3). In terms of substitution, this finding accorded with the preceding study [5] where a substitution rate of 12% v/v at comparable grape TSS levels resulted in inferior colour parameters, and seemed to confirm the implication of a higher sensitivity of Shiraz wine colour to this alcohol management approach, at least in comparison to Cabernet Sauvignon [1,5]. The impact on colour measures was especially apparent when diluting the MF musts (MF_D1–D3) to reach similar alcohol levels as in the MF substitution treatments (MF_S1–S3) (Table 3 and Table 4). At the maximum dilution rate in MF_D1 (47.2% v/v, Table 3), colour density, anthocyanin concentration, SO_2_-resistant pigments and total phenolics were lower by 40%–51% compared to the control, whereas the respective substitution treatments resulted in 23%–34% lower values (Table 4). Comparing these percentages versus the control to those presented for the FF wines, it was evident that water implementation had a greater negative impact on wine colour density and formation of stable pigments with fruit of higher maturity. Contrarily, Cabernet Sauvignon colour parameters were found not to be affected by water substitution treatments [1], or were even enhanced under lower grape ripeness conditions [5].

As observed among the FF wines, the lowest addition/substitution rate did not significantly change the tannin concentration in the MF wines, but the higher rates significantly decreased the tannin levels compared to the control (Table 4). This was also evident among the dilution treatments compared to the respective substitution counterparts at similar alcohol levels, and the effect became significant with the highest dilution/substitution rate (MF_D1/S1). The non-significant difference in MF_S2/D2 could again be indicative of an enhanced tannin extraction in the dilution treatment driven by the lower solid–liquid ratio, as explained for the FF treatments. Similarly to the observations in the FF wines, MF_D2 and MF_S1 wines were equal in tannin concentration and MM.

### 2.4. Implications for Wine Sensory Quality

Aside from the incorporation of additives, water implementation into the winemaking process has generally been viewed with scepticism within the wine industry and among consumers, mainly for preconceived associations with poorer wine quality and dilution of important constituents. As elaborated in the current and previous studies, certain changes in wine chemical composition may occur according to the cultivar, such as less favourable colour characteristics, decrease in tannin concentration, and changes to volatile composition [5]. However, it was also apparent that the overall impact on wine sensory profiles was not as stark as the wine compositional modifications may have suggested, for Cabernet Sauvignon and Shiraz wines arising from pre-fermentative substitution with water [2,5].

**Figure 1:**
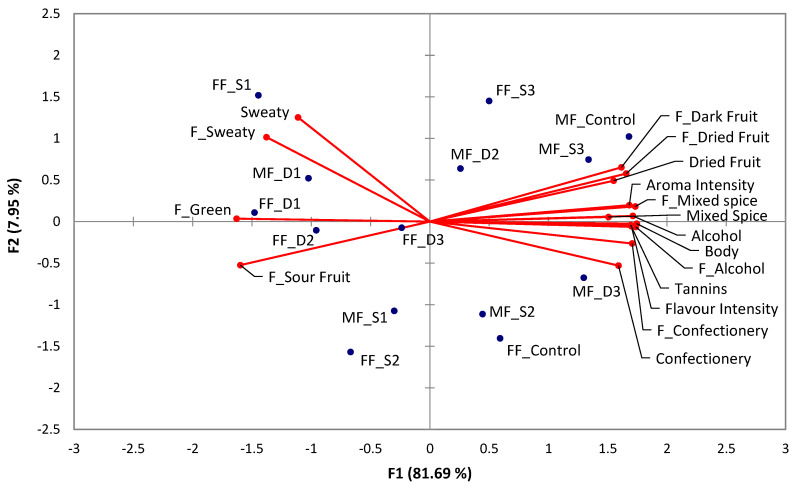
Principal component analysis (PCA) bi-plot of significantly different sensory attributes resulting from the DA panel (Table S2 of the Supporting Information). Sample codes are detailed in Figure S1 of the Supporting Information. The “F” prefix designates flavour attributes.

That prior work was extended upon to examine whether a simple must dilution with water was comparable to the juice substitution option. Based on two-way analysis of variance (ANOVA) of the sensory descriptive analysis (DA) results, only “flavour intensity” or “alcohol” flavour, and “body” and “astringency” differed significantly from the control according to the mode of water implementation, with dilution generally leading to a lower attribute rating compared to substitution (Table 5 and Table 6). The level of water implementation also had an effect of significantly lowering a range of attribute ratings. Comparing the treatments with one-way ANOVA across maturity stages resulted in a total of 17 significantly different attributes, comprising 6 aroma, 9 flavour, and 2 mouthfeel terms (Table S2 of the Supporting Information). These attributes were assessed via principal component analysis (PCA), with the first two principal components presented in the bi-plot in Figure 1 explaining almost 90% of the total variance. Separation occurred mainly along F1, which accounted for 81.69% of the variation, with “sweaty” aroma, and “sweaty”, “green” and “sour fruit” flavours being located opposite to the remaining attributes such related to “alcohol”, “dark fruit” aromas, “confectionery” aroma and flavour, and “flavour intensity”. The control wines made from FF and MF harvests were separated according to both F1 and, by a larger extent, F2. Following the additional twelve days of ripening after the FF harvest date, the MF_Control wine appeared to differ in a number of attributes compared to the FF_Control wine. MF_Control had higher ratings in “alcohol” aroma and flavour, “dried fruit” and “mixed spice” flavour, as well as “body” and “astringency” (Figure 1, Table 5 and Table 6), whereas “sour fruit” flavour declined with the later harvest. This aroma evolution is exemplary for what is usually sought after by winemakers and consumers [26]. The higher “astringency” and “body” perceptions coincided with increments in tannin and glycerol concentrations (Table 3 and Table 4), which could be expected according to previous findings [27,28], whereas the diminishing perception of “sour fruit” aroma (defined as under ripe fruit, Table S1 of the Supporting Information) and flavour was reminiscent of decreasing “green” characteristics in the preceding study [5]. No changes were observed among the majority of sensory descriptors, however, including positive attributes such as “confectionery”, “red fruit”, or “dark fruit”. Thus, even without intervention in the winery by way of water addition, an acceptable wine style might well have been achievable at the FF harvest date in this case, providing a wine with 13.6% ABV compared to the 15.5% ABV wine at the MF stage.

Substituting 11.6% v/v of FF juice with water to produce the FF_S3 wine at 12.6% ABV resulted in a sensory profile that did not significantly differ from the 13.6% ABV FF_Control, except for a higher rating in “sweaty”. In contrast, diluting the juice with an equal amount of water to derive FF_D3 significantly decreased the aroma and flavour perception of “alcohol”, flavour attributes “intensity” and “confectionery”, and “body”, compared to both the control (Table 5). Implementing water via dilution particularly diminished “flavour intensity”, “body” and “astringency” more severely than substitution of a similar proportion of juice. Upon adjusting the alcohol level from 13.5% ABV to around 11% ABV (FF_D2/S2) and further to 9.0% ABV (FF_D1/S1) (Table 3), the wines separated from the FF_Control wine and were generally characterised by more intense “green” and “sour fruit” flavour and “sweaty” aroma characteristics, while lower in desirable attributes like “dried fruit” aroma, “mixed spice” aroma and flavour, ‘‘flavour intensity”, and “body” (Figure 1 and Table 5). The sensory profiles of the pairs of FF_D1/FF_S1 and FF_D2/FF_S2 were similarly perceived (Figure 1) except for a lower “flavour intensity” rating for FF_D1 compared to FF_S1 (Table 5).

The relative sensory similarity between water substitution and dilution treatments remained evident especially at the highest rate as in FF_D1/S1 (Figure 1), with the exception that the dilution treatment resulted in significantly lower “flavour intensity”, whereas this attribute remained similar from FF_S2 to FF_S1 (Table 5). The increased substitution rate from FF_S2 to FF_S1 corresponded to lower “confectionery”, “alcohol” flavour, and “body” ratings, but fruity characters like “red fruit” or “dark fruit” remained similar (Table S2 of the Supporting Information). In contrast, a further dilution from FF_D2 to FF_D1 markedly lowered the “flavour intensity”.

For the riper MF crop, the MF_S3 water substitution treatment affording a 14.5% ABV wine did not significantly differ in sensory quality compared to the MF Control wine (15.5% ABV) except for being lower in “alcohol” flavour and “body”, and neither did dilution with water for MF_D3 (14.4% ABV), except for lower ratings for “mixed spice” aroma and “body” (Table 6). With a decrease in alcohol by approximately 3.5% ABV in wines MF_D2 and MF_S2 (11.7% and 12.0% ABV, respectively), both water implementation treatments continued to have similar sensory profiles, with the exception of lower “body” ratings when dilution was employed. This seemed to drive a separation along component F2 (Figure 1), leaving MF_S2 closely associated with the FF_Control wine. Notably, MF_D2 and MF_S2 wines diverged from the MF_Control (Figure 1) due to lower ratings for “mixed spice” and “alcohol” aroma and flavour, “flavour intensity”, “dried fruit” flavour (except MF_D2), “body” and “astringency” (Table 6).

Sensory profiles for the highest water implementation rate in MF_D1/S1 that afforded wines with 9.6%/10.6% ABV largely remained similar to each other, except for lower perceived “flavour intensity”, “alcohol” flavour, “body” and “astringency” in the MF_D1 wine. This outcome might be at least partially attributed to the alcohol concentration difference of 1% ABV between the treatments (Table 6). Consequently, there was clear separation of both treatments along F1 and F2, positioning MF_D1 closely to FF_D1 (similar % ABV), whereas MF_S1 was more like MF_S2 and FF_Control (Figure 1). Except for the perception of lower “alcohol” flavour, “body”, and “astringency” in MF_S1, the remaining sensory attributes were similar to MF_S2, revealing that the additional decrease of 1.4% ABV from MF_S2 to MF_S1 resulted in a marginal impact on aroma and flavour profiles. It is somewhat remarkable that “red fruit” and “dark fruit” perceptions did not change significantly with up to 47% v/v of water in the must, and ratings for “aroma” and “flavour” intensities seemed not to decline to an extent that this treatment would suggest, at least in the case of substitution (Table S2 of the Supporting Information). Volatile compounds that might be responsible for providing an aroma foundation may have buffered against a more severe “dilution” effect in this case [29].

The highest extent of water substitution in the preceding study [5] was 25% v/v for Shiraz grapes from the same vineyard and of a similar sugar ripeness to the present MF harvest. In line with that study, water substitution at 10.2% v/v (Table 1 and Figure S1 of the Supporting Information) in MF_S3 was inconsequential to wine sensory properties, a finding that generally also applied to dilution giving MF_D3 (Table 6). However, instituting water at 34% v/v as in MF_S2/D2 tended to markedly change the sensory profiles compared to the control—again almost equally for substitution and dilution—except for the notable difference in “body” with dilution (Table 6), which showed strong correlations with decreasing levels of % ABV, tannins, glycerol and total phenolics (r = 0.96, 0.92, 0.91, 0.95, respectively). Although the evidence is limited, taken together it could be that a sweet spot exists between 25% v/v and 34% v/v water implementation before a significant impact on wine sensory profile becomes discernible for MF wines. When considering the lower technological maturity of the FF treatments (i.e., lower grape TSS and pH levels, higher grape malic acid concentrations and lower wine glycerol content), 26.3% v/v juice substitution with water for FF_S2 was already eliciting lower ratings for important attributes like “flavour intensity” and “dark fruit” flavour as well as “body” and “astringency”. This perhaps implies a water substitution sweet spot between 26.3% v/v and 11.6% v/v (FF_S3) for the less ripe FF treatments. However, this does not hold true for the dilution series, as “flavour intensity” and “body” were already significantly lower in FF_D3 with only 11.6% v/v water addition. Therefore, in a lower grape maturity context, water implementation, and dilution in particular, seem to be less likely to maintain a sensory profile as defined by harvest date in comparison with more mature grapes. In addition, the sensory analysis also showed that 13 of 17 attributes were of similar ratings when comparing MF_D3/S3 (lowest water addition level) with the earlier harvested FF_Control (Figure S1 of the Supporting Information), which reinforces the rather limited benefit of the longer grape hang-time of 12 days, particularly if the resulting crop is meant to then be alcohol adjusted. So despite the slightly lower intensities in “body” and higher “sour fruit” perceptions, which are likely to elicit a shift in wine style, an earlier harvest may have been a more sensible option to adjust the wine alcohol level (i.e., within the legal limit for water addition).

## 3. Materials and Methods

### 3.1. Chemicals

Reagents and reference compounds used for analysis were sourced from Sigma Aldrich (Castle Hill, NSW, Australia) or Alfa Aesar (Ward Hill, MA, USA). Stock solutions of standards were prepared volumetrically in redistilled ethanol and stored at −20 °C and working solutions were kept at 4 °C until required. HPLC grade solvents and analytical grade sodium chloride were sourced from Merck (Kilsyth, Victoria, Australia) and Chem-Supply (Gillman, SA, Australia), respectively. Water for analyses was obtained from a Milli-Q purification system (Millipore, north Ryde, NSW, Australia), and filtered tap water was used for the water blending treatments. Potassium metabisulfite (PMS) was sourced from Vebigarden (Padua, Italy).

### 3.2. Climate Data

This study was undertaken during the 2017 vintage in South Australia. Daily minimum, maximum and average temperatures, total monthly rainfall, and long-term averages (Table 1) were obtained from the Australian Bureau of Meteorology [30], as used in preceding studies [1,5]. Based on this data, the Huglin index for the vintage 2016/2017 was calculated as stated in Tonietto and Carbonneau [11].

### 3.3. Harvest and Winemaking

*Vitis vinifera* L. cv. Shiraz grapes were sourced from a commercial vineyard in McLaren Vale, South Australia (138.521139°E, 35.194167°S), using an identical plot to that used previously [5]. Starting from véraison (i.e., 50% of coloured berries) and twice a week, triplicate lots of 200 berries were randomly sampled from both sides of the canopy to monitor the evolution of grape ripening, targeting two distinct grape maturities once berry sugar accumulation had reached a plateau (increase lower than 3 mg sugar/berry/day, according to the model by Deloire [31]). The first batch of grapes for winemaking, subsequently referred to as Fresh Fruit (FF), was harvested on 8 March at 22.7 °Brix, twelve days after the plateau was reached on 24 February. A second harvest occurred twelve days later than the first, on 20 March at 25.5 °Brix, with this stage being designated Mature Fruit (MF).

At each harvest, approximately 400 kg of grapes were collected, destemmed, crushed and distributed in 20 L plastic buckets. Prior to inoculation and in triplicate, must TSS concentrations were diluted either by substituting proportions of juice with water, or by directly adding water, to target similar wine–alcohol levels. Substitution and dilution rates were calculated as previously reported [3]. All buckets were of similar mass after implementing the treatments, at approximately 18–19 kg. The wines originating from the substitution treatments (i.e., maintaining the original solid–liquid ratio) are further referred to as FF_S1–S3 (from the Fresh Fruit harvest) and MF_S1–S3 (from the Mature Fruit harvest), whereas wines resulting from simple dilution with water are further designated FF_D1–D3 and MF_D1–D3 in the same manner. FF_Control and MF_Control designations refer to untreated control wines for each harvest date, which were also prepared in triplicate (Figure S1 of the Supporting Information). Subsequent inoculation and winemaking procedures were the same as previously applied [1,5] The dry wines (<1 g/L residual sugar) were pressed with a basket press, transferred into 10 L glass demijohns, and stored at 0 °C for stabilisation and conservation until bottling. At bottling, wine pH was adjusted to 3.5 (using 500 g/L aqueous tartaric acid solution) and PMS was added at a rate of 100 mg/L. The bottles were stored at 15 °C until analysis.

### 3.4. Analysis of Basic Chemical Parameters

Wine ethanol concentration was measured using an alcolyser (Anton Paar, Graz, Austria). Juice and wine pH and titratable acidity (TA), expressed as g/L equivalents of tartaric acid, were analysed with a T50 Autotitrator (Mettler Toledo, Thebarton, SA, Australia), with a titration endpoint of pH 8.2 using a 0.33 NaOH solution. Glucose, fructose, glycerol, and malic, tartaric, citric and acetic acids were analysed by HPLC using a previously reported method [32].

### 3.5. Extraction and Isolation of Grape and Wine Tannin

#### 3.5.1. Wine-Like Extraction

Grapes were extracted as previously reported [1,33] to estimate extractable tannin content.

#### 3.5.2. Isolation of Wine Tannin

Wine tannins were isolated by solid-phase extraction using a previously published method [34] with a slight modification to collect tannins as one fraction [35].

### 3.6. Analysis of Tannins and Wine Colour

Tannin concentrations of the wine-like extracts (encompassing skin and seed) and wines were assessed using the methylcellulose precipitable tannin assay (MCP tannin) [36]. Colour density, anthocyanin concentration, and total phenolics of wines were analysed using the modified Somers colour assay [36]. Wine tannin size distribution (molecular mass, MM) was determined by gel permeation chromatography (GPC) using methanolic solutions of isolated tannins diluted 1:5 with the HPLC mobile phase prior to injection. Instrument parameters, chromatographic conditions and calibrations for GPC were adapted from Kennedy and Taylor [37] with modifications according to Bindon, Bacic and Kennedy [20].

### 3.7. Sensory Analysis

Wine sensory assessment was conducted via descriptive analysis (DA) ten months after bottling. The DA panel comprised seven female and two male students and researchers from the University of Adelaide that were recruited as assessors because of their previous DA experience. Following the consensus-based approach [38], the DA consisted of nine training and three formal sessions. The panel initially evaluated aroma, flavour and palate characteristics of a subset of wines, which were discussed during two sessions to reach consensus about the descriptive attributes. In subsequent sessions, panellists were given reference standards, that they tried and agreed upon, to familiarise themselves with the aroma attributes as well as mouthfeel characteristics (alcohol, acidity, astringency, bitterness), and further practised with different experimental wines. The wines were rated using RedJade online software and the results obtained from the training sessions were presented to the panellists to provide feedback and screen out non-discriminating attributes. The final attributes list (Table S1 of the Supporting information) included 10 aroma, 10 flavour and 3 mouthfeel attributes, which were rated on 15-cm unstructured line scales, with anchors at 10%, 50% and 90% of the scale corresponding to “low”, “medium” and “high”, respectively. During the formal assessments, panellists were presented 14 wine samples (30 mL) served in ISO standard (ISO 3951:1977) black wine glasses coded with four-digit numbers and covered with glass lids in a randomised and balanced order. The evaluations were held in a sensory laboratory equipped with isolated booths, under an ambient temperature of 21 °C, and with white lighting. One min rest breaks after each sample and five min after seven samples were imposed on the panellists to avoid fatigue. Pectin solution (1 g/L, pectin from citrus peel, Sigma-Aldrich, Castle Hill, NSW, Australia), plain water crackers and filtered tap water were provided to the panellists for palate cleansing. The sensory study was approved by the Human Research Ethics Committee of the University of Adelaide (Approval No. H-2015-211).

### 3.8. Statistical Analysis

One-way (all treatments) and two-way (mode of water addition and level of dilution, separately for each maturity stage) analysis of variance (ANOVA) of the chemical and sensory data in combination with mean comparisons via Fisher’s least significant difference (LSD) multiple comparison test at α = 0.05, and principal component analysis (PCA) of normalised significant sensory data (one-way ANOVA) were performed using XLSTAT (Version 2015.4.1, Addinsoft, Paris, France). PanelCheck (V1.4.2, Nofima Mat, Norway) was used to assess panel performance during the DA, and the final results were analysed via ANOVA and mean comparisons by Fisher’s LSD using SENPAQ (Version 6.03, Qi Statistics, Reading, Berkshire, United Kingdom).

## 4. Conclusions

This study builds on preceding observations that pre-fermentative juice substitution with a proportionate amount of water (within certain limits) appears to be a suitable approach to controlling alcohol concentrations in Cabernet Sauvignon and Shiraz wines while maintaining wine chemical and sensory characteristics as defined by the harvest date. The approach was extended to the simple dilution of must with water, which is likely to be a preferred method given the easier implementation (while accounting for potential volume losses through berry shrivel). Furthermore, the implications of grape technological maturity on Shiraz wine quality were considered with the blending treatments. At a lower grape maturity as in the FF treatments, juice substitution of 11.6% v/v with water did not change colour properties in contrast to analogous treatment involving dilution with water, which revealed declines in colour intensity and stable forms of colour in line with total phenolics and tannin concentrations. The impact of dilution was further mirrored in a decline in important sensory attributes such as “flavour intensity” and “body” and the divergence between pre-fermentative substitution and dilution became more obvious with lower established alcohol levels (i.e., more water added).

With riper grapes used for the MF treatments, substituting or diluting the must with 10.2% v/v water decreased wine colour properties, but substitution treatments appeared to have a greater effect than with the less ripe fruit. Tannin concentration remained stable with low substitution and dilution rates alike although notable declines were seen with the higher dilution rates. Nonetheless, wine sensory qualities as determined by the harvest date were more or less maintained, although with higher water implementation rates, the decline in an array of attributes was noticeable. Despite this, the difference between dilution and substitution was less pronounced compared to the case for the grapes picked at a lower technological maturity. Ultimately, managing the alcohol level based on the mature crop (within the legal limit) resulted in a sensory profile reminiscent of the wines from the earlier harvest, negating the presumed benefits of a longer hang-time if alcohol adjustment is desired, regardless. In this case, earlier harvesting could decompress the harvest process and decrease winemaking input, i.e., post-harvest alcohol management.

Overall, the knowledge generated in this study contributes to the ability of winemakers to make informed decisions about the most suitable way for pre-fermentative water addition according to desired wine styles and harvest date while working within the relevant regulations. Winemakers may expect a more detrimental influence on wine flavour and mouthfeel attributes with a dilution approach and decisions around this mode of water inclusion should be made with caution. Much scope still exists for this experimental design to be applied to other cultivars (especially in warm climate viticultural regions), given the apparent dependence of variety on the suitability of this approach, as now observed across a number of studies.

## Figures and Tables

**Table 1:** Weather conditions near the McLaren Vale ^1^ vineyard during the 2016/2017 growing season. Minimum (Ø Temp Min), maximum (Ø Temp Max) and average (Ø Temp) temperatures, as well as precipitation sums (∑ Rainfall) are compared to the long-term values (2000–2017, in parentheses). Numbers in bold indicate values above (for temperature) or below (for rainfall) the average values.

Month	Ø Temp Min (°C)	Ø Temp Max (°C)	Ø Temp (°C)	∑ Rainfall (mm)
Jul ′16	**9.0 (+0.3)**	14.5 (−0.3)	11.8 (0.0)	118.4 (+51.8)
Aug ′16	8.8 (0.0)	**16.5 (+0.6)**	**12.7 (+0.3)**	68.6 (+14.9)
Sep ′16	9.8 (−0.6)	16.6 (−2.0)	13.2 (−1.3)	64.0 (+18.1)
Oct ′16	10.4 (−1.1)	19.9 (−1.5)	15.2 (−1.3)	65.8 (+33.8)
Nov ′16	12.0 (−2.1)	22.8 (−2.1)	17.4 (−2.1)	**16.0 (−5.1)**
Dec ′16	15.2 (−0.2)	**26.6 (+0.2)**	20.9 (0.0)	48.0 (+26.1)
Jan ′17	**17.6 (+0.5)**	28.5 (−0.2)	**23.1 (+0.2)**	34.2 (+16.1)
Feb ′17	16.5 (−0.4)	26.8 (−0.9)	21.7 (−0.6)	**14.2 (−5.4)**
Mar ′17	**17.5 (+1.9)**	27.7 (+2.0)	**22.6 (+1.9)**	**9.2 (−12.4)**
Apr ′17	**14.1 (+0.5)**	22.5 (+0.2)	**18.3 (+0.3)**	50.0 (+16.6)
May ′17	11.1 (−0.4)	18.3 (−0.2)	14.7 (−0.3)	**25.8 (−27.9)**
Jun ′17	7.7 (−1.6)	**16.2 (+0.6)**	12.0 (−0.5)	**21.8 (−40.9)**

^1^ Noarlunga weather station (Latitude: 35.16 °S, Longitude 138.51 °E, Elevation: 55 m).

**Table 2:** Basic grape compositional parameters at two distinct harvest dates (Fresh Fruit (FF) and Mature Fruit (MF)) and grape extractable tannin.^1^

Parameter	Fresh Fruit	Mature Fruit
Harvest date	8 March 2017	20 March 2017
TSS [°Brix]	22.7 ± 0.1 ^b^	25.5 ± 0.0 ^a^
Berry weight [g/berry]	0.89 ± 0.02	0.90 ± 0.02
TA [g/L] ^2^	5.8 ± 0.1 ^a^	4.5 ± 0.1 ^b^
pH	3.82 ± 0.00 ^b^	4.03 ± 0.00 ^a^
*Extractable tannin* ^3^		
mg/g berry	0.36 ± 0.01 ^b^	0.53 ± 0.02 ^a^
mg/berry	0.31 ± 0.01 ^b^	0.38 ± 0.03 ^a^

^1^ Values are means of three replicates ± standard error. Different superscripted lower-case letters within rows indicate significantly different means (*p* ≤ 0.05, one-way analysis of variance (ANOVA)). ^2^ Values in tartaric acid equivalents. ^3^ Determined by methylcellulose precipitable tannin assay of wine-like extracts for crushed berries.

**Table 3:** Water addition rates, basic chemical parameters, and p-values for wines resulting from the Fresh Fruit (FF_Control, FF_S and FF_D series) and Mature Fruit (MF_Control, MF_S and MF_D series) harvests.^1^

Wine	Water Addition Rate [% v/v]	Alcohol Level [% v/v]	TA [g/L] ^2^	pH	Malic Acid [g/L]	Acetic Acid [g/L]	Glycerol [g/L]	Acetaldehyde [g/L]	Fructose [g/L] ^3^
FF Control	n/a	13.6 ± 0.1 ^a^	7.1 ± 0.2	3.48 ± 0.05 ^c^	3.05 ± 0.65	0.37 ± 0.04 ^a,b^	9.0 ± 1.22 ^a,b^	0.46 ± 0.17 ^a^	0.11 ± 0.16
FF_S1	41.0	9.6 ± 0.1 ^e^	6.5 ± 0.1	3.53 ± 0.01 ^b,c^	2.83 ± 0.08	0.42 ± 0.02 ^a^	7.2 ± 0.1 ^c,d^	0.26 ± 0.01 ^b,c^	0.10 ± 0.14
FF_S2	26.3	11.1 ± 0.1 ^c^	6.7 ± 0.1 ^b^	3.58 ± 0.05 ^a,b^	3.07 ± 0.03	0.36 ± 0.01 ^b,c^	8.1 ± 0.2 ^b,c^	0.32 ± 0.01 ^a,b,c^	0.22 ± 0.16
FF_S3	11.6	12.6 ± 0.0 ^b^	7.0 ± 0.0	3.57 ± 0.01 ^a,b,c^	3.36 ± 0.17	0.32 ± 0.01 ^c,d^	9.2 ± 0.2 ^a^	0.40 ± 0.06 ^a,b^	0.12 ± 0.18
FF_D1	41.0	9.0 ± 0.1 ^f^	6.2 ± 0.0	3.39 ± 0.03 ^d^	2.52 ± 0.03	0.39 ± 0.00 ^a,b^	6.7 ± 0.1 ^d^	0.21 ± 0.01 ^c^	0.08 ± 0.11
FF_D2	26.3	10.8 ± 0.1 ^d^	6.6 ± 0.1 ^b^	3.53 ± 0.03 ^b,c^	2.94 ± 0.06	0.34 ± 0.04 ^b,c,d^	8.0 ± 0.2 ^c^	0.28 ± 0.03 ^b,c^	0.20 ± 0.14
FF_D3	11.6	12.6 ± 0.1 ^b^	7.9 ± 1.4	3.62 ± 0.02 ^a^	3.25 ± 0.06	0.30 ± 0.02 ^d^	9.1 ± 0.1 ^a,b^	0.38 ± 0.03 ^a,b^	0.12 ± 0.17
*p*-value	-	<0.0001	0.120	<0.0001	0.087	0.004	0.000	0.039	0.951
Control	-	13.6 ^a^	7.1	3.48 ^a^	3.05	0.37	9.0a	0.46	0.11
Substitution	-	11.1 ^b^	6.8	3.56 ^b^	3.09	0.36	8.2b	0.33	0.15
Dilution	-	10.8 ^c^	6.9	3.51 ^c^	2.90	0.34	7.9b	0.29	0.13
*p*-value	-	<0.0001	0.6332	0.0138	0.2251	0.1013	0.3081	0.3516	0.8680
MF Control	n/a	15.5 ± 0.1 ^a^	7.0 ± 0.0 ^b^	3.52 ± 0.01 ^a^	2.86 ± 0.02 ^a^	0.33 ± 0.03	11.6 ± 0.1 ^a^	0.75 ± 0.00 ^a^	0.90 ± 0.29 ^a^
MF_S1	47.2	10.6 ± 0.1 ^e^	6.6 ± 0.2 ^c^	3.50 ± 0.00 ^a,b^	2.28 ± 0.07 ^b,c^	0.38 ± 0.01	7.6 ± 0.1 ^d^	0.33 ± 0.03 ^c,d^	0.39 ± 0.01 ^b^
MF_S2	34.0	12.0 ± 0.0 ^c^	6.7 ± 0.0 ^c^	3.49 ± 0.02 ^b,c^	2.47 ± 0.05 ^b^	0.35 ± 0.04	8.6 ± 0.2 ^c,d^	0.36 ± 0.02 ^c^	0.44 ± 0.01 ^b^
MF_S3	10.2	14.5 ± 0.0 ^b^	7.3 ± 0.2 ^a^	3.52 ± 0.02 ^a^	2.76 ± 0.02 ^a^	0.32 ± 0.0	10.5 ± 0.1 ^b^	0.61 ± 0.03 ^b^	0.58 ± 0.01 ^b^
MF_D1	47.2	9.60 ± 0.20 ^f^	6.3 ± 0.0 ^d^	3.31 ± 0.02 ^e^	2.17 ± 0.19 ^c^	0.30 ± 0.02	7.8 ± 0.5 ^d^	0.23 ± 0.04 ^d^	0.35 ± 0.05 ^b^
MF_D2	34.0	11.7 ± 0.1 ^d^	6.5 ± 0.0 ^c^^,^^d^	3.38 ± 0.00 ^d^	2.47 ± 0.24 ^b^	0.31 ± 0.05	9.1 ± 1.0 ^c^	0.36 ± 0.14 ^c^	0.46 ± 0.08 ^b^
MF_D3	10.2	14.4 ± 0.0 ^b^	7.0 ± 0.1 ^b^	3.46 ± 0.02 ^c^	2.78 ± 0.01 ^a^	0.29 ± 0.01	10.7 ± 0.2 ^a,b^	0.55 ± 0.02 ^b^	0.54 ± 0.00 ^b^
*p*-value		<0.0001	<0.0001	<0.0001	<0.0001	0.074	<0.0001	<0.0001	<0.0001
Control	-	15.5 ^a^	7.0	3.52 ^a^	2.85	0.35 ^a^	11.6	0.75	0.90
Substitution	-	12.4 ^b^	6.8 ^a^	3.50 ^a^	2.50	0.33 ^a,b^	8.9	0.44	0.47
Dilution	-	11.9 ^c^	6.6 ^b^	3.39 ^b^	2.47	0.30 ^b^	9.2	0.38	0.45
*p*-value	-	<0.0001	0.0033	<0.0001	0.6639	0.0055	0.2955	0.1126	0.7488

^1^ Values are means of three replicates ± standard error. Different superscripted lower-case letters within columns indicate significantly different means (*p* ≤ 0.05, one-way (treatment) and two-way (mode [shown with grey shading] and level of water addition) ANOVA) within a given maturity stage.^2^ Values in tartaric acid equivalents. ^3^ Values also correspond to total sugars as glucose was entirely consumed.

**Table 4:** Colour, phenolic, and tannin parameters and p-values for wines resulting from Fresh Fruit (FF_Control, FF_S and FF_D series) and Mature Fruit (MF_Control, MF_S and MF_D series) harvests.^1^

Wine	Colour Density [au]	Total Anthocyanin [mg/L]	Total Phenolics [au]	SO_2_ Resistant Pigments [au]	Tannin MM [g/mol] ^2^	MCP Tannin [mg/L] ^3^
FF Control	8.7 ± 0.2 ^a^	693 ± 11 ^a^	42.8 ± 0.8 ^a^	1.42 ± 0.06 ^a^	1544 ± 22 ^a^	595 ± 48 ^a,b^
FF_S1	7.7 ± 0.2 ^c^	598 ± 12 ^c^	36.0 ± 0.8 ^c^	1.16 ± 0.02 ^c^	1489 ± 4 ^b^	463 ± 37 ^b,c^
FF_S2	8.2 ± 0.4 ^b^	654 ± 15 ^b^	39.4 ± 0.8 ^b^	1.25 ± 0.03 ^b^	1516 ± 13 ^a,b^	399 ± 23 ^c^
FF_S3	8.9 ± 0.2 ^a^	688 ± 17 ^a^	41.9 ± 0.7 ^a^	1.39 ± 0.04 ^a^	1534 ± 18 ^a^	651 ± 1 ^a^
FF_D1	5.8 ± 0.1 ^e^	468 ± 9 ^e^	27.8 ± 0.9 ^e^	0.89 ± 0.01 ^e^	1421 ± 18 ^c^	231 ± 17 ^d^
FF_D2	6.8 ± 0.1 ^d^	540 ± 20 ^d^	32.7 ± 0.8 ^d^	1.08 ± 0.01 ^d^	1477 ± 31 ^b^	455 ± 80 ^b,c^
FF_D3	8.6 ± 0.1 ^a,b^	647 ± 12 ^b^	40.1 ± 1.0 ^b^	1.30 ± 0.02 ^b^	1537 ± 10 ^a^	618 ± 148 ^a^
*p*-value	<0.0001	<0.0001	<0.0001	<0.0001	<0.0001	<0.0001
Control	8.7 ^a^	693 ^a^	42.8 ^a^	1.42 ^a^	1544 ^a^	595
Substitution	8.3 ^b^	647 ^b^	39.1 ^b^	1.27 ^b^	1513 ^a^	504
Dilution	7.1 ^c^	552 ^c^	33.5 ^c^	1.09 ^c^	1478 ^b^	435
*p*-value	<0.0001	<0.0001	<0.0001	<0.0001	0.0055	0.0987
MF Control	15.4 ± 0.5 ^a^	944 ± 33 ^a^	61.0 ± 2.0 ^a^	2.30 ± 0.05 ^a^	1640 ± 47 ^a^	917 ± 61 ^a^
MF_S1	10.2 ± 0.3 ^e^	727 ± 10 ^e^	42.6 ± 0.8 ^e^	1.52 ± 0.03 ^e^	1509 ± 10 ^b,c^	680 ± 62 ^b^
MF_S2	11.7 ± 0.1 ^d^	784 ± 10 ^d^	47.0 ± 0.7 ^d^	1.71 ± 0.01 ^d^	1551 ± 11 ^b^	734 ± 62 ^b^
MF_S3	14.2 ± 0.2 ^b^	905 ± 19 ^b^	57.2 ± 0.6 ^b^	2.16 ± 0.03 ^b^	1639 ± 18 ^a^	1011 ± 67 ^a^
MF_D1	7.80 ± 0.19 ^f^	565 ± 4 ^g^	33.5 ± 0.3 ^f^	1.12 ± 0.01 ^f^	1492 ± 3 ^c^	286 ± 20 ^c^
MF_D2	11.1 ± 1.2 ^d,e^	673 ± 18 ^f^	42.0 ± 0.7 ^e^	1.50 ± 0.07 ^e^	1536 ± 6 ^b,c^	598 ± 109 ^b^
MF_D3	12.8 ± 0.3 ^c^	821 ± 6 ^c^	50.4 ± 0.5 ^c^	1.93 ± 0.03 ^c^	1633 ± 16 ^a^	951 ± 43 ^a^
*p*-value	<0.0001	<0.0001	<0.0001	<0.0001	<0.0001	<0.0001
Control	15.4 ^a^	944 ^a^	61.0 ^a^	2.30 ^a^	1640	917 ^a^
Substitution	12.0 ^b^	806 ^b^	48.9 ^b^	1.80 ^b^	1566	809 ^a^
Dilution	10.6 ^c^	686 ^c^	42.0 ^c^	1.51 ^c^	1554	612 ^b^
*p*-value	0.0003	<0.0001	<0.0001	<0.0001	0.3053	<0.0001

^1^ Values are means of three replicates ± standard error. Different superscripted lower-case letters within columns indicate significantly different means (*p* ≤ 0.05, one-way (treatment) and two-way (mode [shown with grey shading] and level of water addition) ANOVA) within a given maturity stage. ^2^ Molecular mass determined by gel permeation chromatography at 50% elution. ^3^ Determined by methylcellulose precipitable (MCP) tannin assay.

**Table 5:** Average scores and *p*-values for significantly different (*p* < 0.05) sensory attributes of the wines based on the Fresh Fruit (FF) harvest.^1^

		Fresh Fruit										
	FF Control	FF_D1	FF_D2	FF_D3	FF_S1	FF_S2	FF_S3	*p*-value	Control	Dilution	Substi-tution	*p*-Value
% *ABV*	13.6	9.0	10.8	12.6	9.6	11.1	12.6	-	-	-	-	-
*Aroma*												
Dried fruit	42.1 ^a,b^	26.2 ^c^	33.6 ^a,b,c^	48.0 ^a,b^	29.0 ^b,c^	29.3 ^b,c^	50.4 ^a^	0.0174	42.1	35.9	34.5	0.949
Confectionery	49.2 ^a^	27.1 ^c,d^	31.6 ^b,c,d^	37.7 ^a,b,c^	20.5 ^d^	40.3 ^a,b,c^	46.3 ^a,b^	0.0038	49.2	32.1	34.4	0.444
Mixed spice	45.2 ^a^	22.8 ^c^	23.4 ^c^	38.6 ^a,b,c^	27.1 ^b,c^	29.3 ^b,c^	35.3 ^a,b,c^	0.0035	45.2	28.3	30.0	0.552
Sweaty	26.7 ^c^	47.1 ^a,b^	44.0 ^a,b^	34.8 ^b,c^	57.0 ^a^	36.2 ^b,c^	50.2 ^a,b^	0.0039	26.7	41.9	47.5	0.215
Alcohol	51.3 ^a^	29.2 ^c^	35.0 ^b,c^	36.1 ^b,c^	30.0 ^c^	43.8 ^a,b,c^	51.1 ^a,b^	0.0145	51.3	33.4	40.4	0.074
*Flavour*												
Flavour intensity	65.1 ^a^	35.3 ^d^	51.8 ^bc^	52.3 ^b,c^	47.7 ^c^	51.0 ^c^	63.8 ^a,b^	<0.0001	65.1 ^a^	46.4 ^c^	53.0 ^b^	0.020
Sour fruit	48.3 ^b,c^	57.3 ^a,b^	66.7 ^a^	54.8 ^a,b,c^	63.4 ^a^	67.0 ^a^	40.2 ^c^	0.0073	48.3	59.6	59.0	0.541
Dried fruit	33.8 ^a,b^	20.1 ^b^	25.1 ^b^	31.6 ^a,b^	21.5 ^b^	19.6 ^b^	45.3 ^a^	0.0194	33.8	25.6	26.7	0.474
Dark fruit	52.1 ^a,b^	35.8 ^b,c^	39.7 ^b,c^	48.5 ^a,b,c^	41.0 ^b,c^	33.0 ^c^	63.6 ^a^	0.0187	52.1	41.3	43.6	0.374
Confectionery	47.4 ^a^	16.8 ^d^	26.3 ^c,d^	29.7 ^b,c^	15.8 ^d^	32.6 ^b,c^	40.4 ^a,b^	<0.0001	47.4	24.2	28.2	0.161
Mixed spice	36.6 ^a^	18.8 ^d^	21.5 ^c,d^	32.8 ^a,b,c^	23.5 ^d,c,d^	21.9 ^c,d^	36.1 ^a,b^	0.0055	36.6	24.4	26.0	0.420
Green	29.8 ^c^	44.9 ^a^	42.5 ^a,b^	39.0 ^a,b,c^	48.3 ^a^	38.3 ^a,b,c^	23.3 ^c^	0.0402	29.8	42.1	38.3	0.227
Alcohol	62.8 ^a^	23.8 ^d^	35.3 ^c,d^	47.5 ^b,c^	26.6 ^d^	41.6 ^b,c^	55.3 ^a,b^	<0.0001	62.8	35.5	39.4	0.145
*Mouthfeel*												
Body	53.3 ^a^	20.4 ^c^	29.3 ^b,c^	37.3 ^b^	25.3 ^c^	37.0 ^b^	50.8 ^a^	<0.0001	53.3 ^a^	29.0 ^c^	36.0 ^b^	0.009
Astringency	53.5 ^a^	22.0 ^e^	38.3 ^cd^	43.0 ^a,b,c^	30.5 ^d,e^	40.9 ^b,c,d^	52.8 ^a,b^	<0.0001	53.5 ^a^	34.4 ^b^	40.0 ^b^	0.042

^1^ Values are means of three replicates. Different superscripted lower-case letters within a row indicate significantly different means (*p* ≤ 0.05, one-way (treatment) and two-way (mode [shown with grey shading] and level of water addition) ANOVA, post hoc Fisher’s least significant difference (LSD)) within a given maturity stage.

**Table 6:** Average scores and *p*-values for significantly different (*p* < 0.05) sensory attributes of the wines based on the Mature Fruit (MF) harvest.^1^

		Mature Fruit										
	MF Control	MF_D1	MF_D2	MF_D3	MF_S1	MF_S2	MF_S3	*p*-value	Control	Dilution	Substi-tution	*p*-Value
% *ABV*	15.5	9.6	11.7	14.4	10.6	12.0	14.5	-	-	-	-	-
*Aroma*												
Aroma intensity	71.3 ^a^	52.5 ^c^	62.8 ^a,b^	69.7 ^a^	58.4 ^b,c^	59.0 ^b,c^	69.0 ^a^	0.0005	71.3	61.7	62.2	0.857
Dried fruit	52.2 ^a,b^	40.0 ^b,c^	47.9 ^a,b^	52.5 ^a,b^	30.0 ^c^	44.8 ^a,b,c^	58.2 ^a^	0.0245	52.2	46.8	44.3	0.603
Confectionery	50.8	28.5	40.0	46.0	40.8	42.6	42.5	0.1701	50.8	38.5	42.0	0.448
Mixed spice	50.8 ^a^	30.0 ^c^	34.9 ^b,c^	35.1 ^b,c^	37.7 ^a,b,c^	33.3 ^b,c^	46.3 ^a,b^	0.0221	50.8	33.3	39.1	0.134
Sweaty	36.7	43.2	40.2	26.2	36.1	22.0	32.3	0.0894	36.7	36.5	30.1	0.159
Alcohol	69.5 ^a^	29.8 ^d^	49.8 ^b,c^	65.0 ^a^	37.5 ^c,d^	44.4 ^c^	61.6 ^a,b^	<0.0001	69.5	48.2	47.8	0.930
*Flavour*												
Flavour intensity	75.3 ^a^	40.9 ^d^	56.5 ^c^	71.7 ^a,b^	54.5 ^c^	63.9 ^b,c^	67.5 ^a,b^	<0.0001	75.3	56.4	62.0	0.058
Sour fruit	28.0 ^c^	53.5 ^a^	37.8 ^b,c^	36.5 ^b,c^	51.3 ^a,b^	45.8 ^a,b^	29.0 ^c^	0.0028	29.0	42.6	42.0	0.893
Dried fruit	58.5 ^a^	30.4 ^c,d^	45.0 ^a,b,c^	50.9 ^a,b^	25.9 ^d^	38.8 ^b,c,d^	54.8 ^a,b^	0.0003	58.5	42.1	39.8	0.633
Dark fruit	68.5 ^a^	45.9 ^b,c^	53.3 ^a,b,c^	60.8 ^a,b^	40.9 ^c^	55.4 ^a,b,c^	66.0 ^a^	0.0069	68.5	53.3	54.1	0.862
Confectionery	49.8 ^a^	20.5 ^c^	33.8 ^b,c^	47.5 ^a,b^	34.2 ^c^	34.0 ^b,c^	50.8 ^a^	0.0001	49.8	33.9	38.7	0.259
Mixed spice	50.4 ^a^	22.4 ^c^	33.0 ^b,c^	42.0 ^a,b^	31.6 ^b,c^	38.4 ^a,b^	46.2 ^a^	0.0005	50.4	32.4	38.7	0.098
Green	19.2 ^c^	34.5 ^a^	23.1 ^b,c^	18.6 ^c^	30.4 ^a,b^	23.0 ^b,c^	24.2 ^a,b,c^	0.0442	19.2	25.4	25.9	0.886
Alcohol	83.0 ^a^	19.8 ^e^	48.6 ^c^	74.5 ^a,b^	36.3 ^d^	57.3 ^c^	72.9 ^b^	<0.0001	83 ^a^	47.7 ^c^	55.5 ^b^	0.006
*Mouthfeel*												
Body	74.7 ^a^	21.8 ^e^	38.8 ^d^	63.3 ^b^	38.3 ^d^	50.0 ^c^	64.3 ^b^	<0.0001	74.7 ^a^	41.3 ^c^	50.8 ^b^	0.0003
Astringency	66.5 ^a^	20.0 ^d^	44.1 ^b,c^	60.9 ^a^	35.8 ^c^	50.1 ^b^	60.6 ^a^	<0.0001	66.5 ^a^	41.7 ^c^	48.8 ^b^	0.010

^1^ Values are means of three replicates. Different superscripted lower-case letters within a row indicate significantly different means (*p* ≤ 0.05, one-way (treatment) and two-way (mode [shown in grey shading] and level of water addition) ANOVA, post hoc Fisher’s LSD) within a given maturity stage.

## Supplementary Materials

The following are available online, Table S1: Attribute list used in descriptive analysis, Figure S1: Attribute list used in descriptive analysis, Table S2: All ratings as determined by the descriptive analysis panel.
