# Supplementary material for: Substitution or Dilution? Assessing Pre-Fermentative Water Implementation to Produce Lower Alcohol Shiraz Wines
Source: Molecules. 2020 May 10;25(9):2245. doi: 10.3390/molecules25092245 (PMC7249075; doi:10.3390/molecules25092245)
Supplement: Supplementary file 1 [file molecules-25-02245-s001.pdf]

SUPPORTING INFORMATION FOR

Substitution or Dilution? Assessing Pre-fermentative Water Implementation to Produce Lower Alcohol Shiraz Wines

Olaf J. Schelezki [1,2,†], Alain Deloire [3] and David W. Jeffery [1,2*]

[1] Department of Wine and Food Science, and Waite Research Institute, The University of Adelaide (UA), Waite Campus, PMB 1, Glen Osmond, SA 5064, Australia
[2] Australian Research Council Training Centre for Innovative Wine Production, UA, Waite Campus, PMB 1, Glen Osmond, SA 5064, Australia
[3] Department of Biology-Ecology, University of Montpellier, L'institut Agro (SupAgro), 2 Place Pierre Viala, 34060 Montpellier, France
* Correspondence: david.jeffery@adelaide.edu.au; Tel.: +61-8-8313-6649
† Present address: Faculty of Agriculture and Life Sciences, Lincoln University, PO Box 85084, Lincoln 7647, Canterbury, New Zealand

Table of Contents

Page

Table S1. Attribute list developed by the descriptive analysis panel to describe and rate the wine sensory profiles, showing respective definitions and aroma/flavour reference standards. S-2

Figure S1. Flowchart presenting the main wine production steps and resulting treatment definitions, water implementation rates and final wine alcohol levels. S-3

Table S2. Mean ratings for sensory attributes that were rated by the descriptive analysis panel, including p-values and least significant difference values (LSD) for Fresh Fruit (FF) and Mature Fruit (MF) wines. S-4

Table S1. Attribute list developed by the descriptive analysis panel to describe and rate the wine sensory profiles, showing respective definitions and aroma/flavour reference standards.

Descriptor	Type	Definition	Standard mixed in 30 mL of Shiraz cask wine (Yalumba, 2017)
Dark fruit	Aroma/Flavour	Fresh blackcurrants, mulberry, blueberry	2 pieces of crushed blackberry (Sunnyside); 4 fresh blueberries, cut; 7 mL blackcurrant cordial (Bickford's), 7 mL blackcurrant juice (Ribena)
Red fruit	Aroma/Flavour	Fresh raspberry, strawberry, red currents	1 cm cube of fresh strawberry and 1 cm cube of fresh raspberry
Confectionery	Aroma/Flavour	Sweet lolly	1 strawberry and cream lolly, cut (5 g) (Allens)
Sour fruit	Aroma/Flavour	Under ripe fruit, fresh tart plum; sour cherries	2 cm fresh plum, cut, 4 crushed pitted sour cherries and 6 mL of cherry juice (Takoland)
Dried fruit	Aroma/Flavour	Over ripe fruit or cooked fruit, plum jam, prune, raisins	1 tablespoon plum jam (Cottee's), 7 dried sultanas, cut (Nature's Delight), 1 pitted prune, crushed (Nature's Delight)
Mixed spice	Aroma/Flavour	Sweet spice, including cinnamon, nutmeg, cloves and liquorice	¼ teaspoon of ground allspice (Coles) + 1 piece liquorice, cut (Marco Polo)
Savoury	Aroma/Flavour	Salty; soy sauce; medicinal	5 mL of tamari oyster sauce (Chang's)
Green	Aroma/Flavour	Stalky, grassy, granny smith apple peel	2 cm fresh grapevine stalks and ½ grape vine leaf, cut
Sweaty	Aroma/Flavour	Sweat horse, dirty, dusty, earthy, 'bretty' (odour description associated with *Brettanomyces bruxellensis*)	5 mL of Merlot wine inoculated with *Brettanomyces bruxellensis* (AWRI 1499)
Alcohol	Aroma/Flavour	Level of alcohol perceived	3 mL of 70% ethanol
Acidity	Mouthfeel	Level of acid perceived	Tartaric acid (low, 0.5 g/L; high, 2 g/L)
Bitterness	Mouthfeel	Perception of bitterness	quinine sulfate (low, 5 mg/L; high, 20 mg/L)
Astringency	Mouthfeel	Perception of drying or puckering sensation	Felt material (low), sandpaper (high)

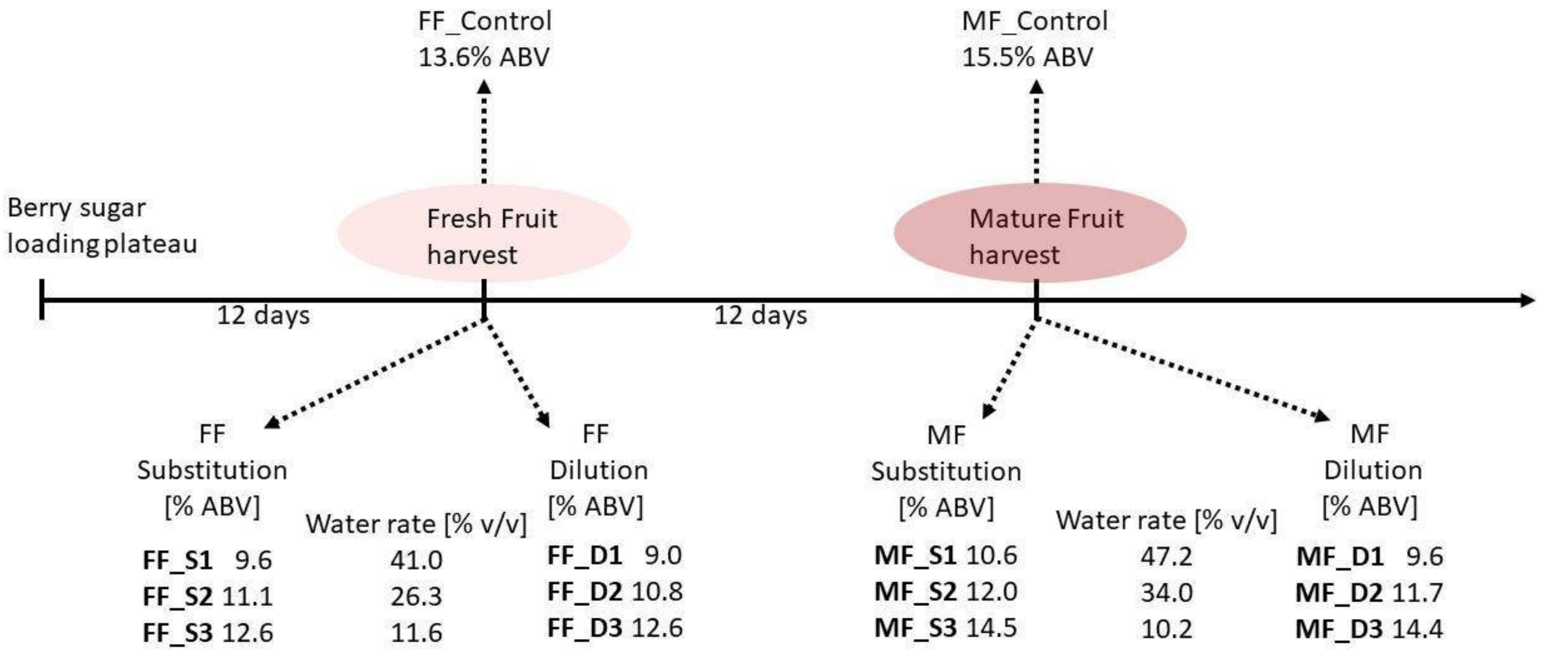

Figure S1. Flowchart presenting the main wine production steps and resulting treatment definitions, water implementation rates and final wine alcohol levels.

Table S2. Mean ratings for sensory attributes that were rated by the descriptive analysis panel, including p-values and least significant difference values (LSD) for Fresh Fruit (FF) and Mature Fruit (MF) wines.[1]

	Fresh Fruit							*Mature Fruit*								
	FF Control	FF_D1	FF_D2	FF_D3	FF_S1	FF_S2	FF_S3	MF Control	MF_D1	MF_D2	MF_D3	MF_S1	MF_S2	MF_S3	p-value	LSD
% ABV	*13.6*	*9*	*10.8*	*12.6*	*9.6*	*11.1*	*12.6*	*15.5*	*9.6*	*11.7*	*14.4*	*10.6*	*12*	*14.5*		
Aroma																
Aroma intensity	58.1cd	52.5d	55.5cd	56.6cd	51.6d	55.8cd	60.6bcd	71.3a	52.5d	62.8abc	69.7ab	58.4cd	59cd	69ab	0.0001	9.5
Sour fruit	42.0	45.3	47.3	32.5	46.4	53.5	32.1	31.1	38.0	33.1	30.5	46.7	36.1	32.3	0.0980	16.9
Dried fruit	42.1abcde	26.2e	33.6cde	48abc	29de	29.3de	50.4abc	52.2ab	40bcde	47.9abc	52.5ab	30de	44.8abcd	58.2a	0.0010	16.9
Red fruit	48.8	38.8	41.1	45.5	28.2	52.7	35.8	44.1	37.9	41.5	37.7	44.2	39.8	40.c	0.3297	15.5
Dark fruit	43.9	37.1	41.5	48.8	39.3	36.1	52.5	60.5	51.0	49.4	62.4	50	50	54.1	0.0660	17.1
Confectionery	49.2a	27.1cd	31.6bcd	37.7abc	20.5d	40.3abc	46.3ab	50.8a	28.5cd	40abc	46.9ab	40.8abc	42.6abc	42.5abc	0.0054	15.8
Mixed spice	45.2ab	22.8d	23.4d	38.6abc	27.1cd	29.3cd	35.3bcd	50.8a	30cd	34.9bcd	35.1bcd	37.7abc	33.3bcd	46.3ab	0.0006	13.2
Savoury	29.5	43.3	36.8	34.5	41.4	33.8	32.6	39.1	33.2	36.5	29.4	32.4	26.8	35.9	0.7854	15.8
Green	62.5	50.3	59.3	66.7	61.6	57.4	54.8	55.2	46.5	48.8	54.1	38	51	50.7	0.0585	17.2
Sweaty	26.7de	47.1abc	44abc	34.8bcde	57a	36.2bcde	50.2ab	36.7bcde	43.2abcd	40.2abcd	26.2de	36.1bcde	22e	32.3cde	0.0051	12.8
Alcohol	51.3bc	29.2f	35ef	36.1ef	30f	43.8cde	51.1bc	69.5a	29.8f	49.8bcd	65a	37.5def	44.4cde	61.6ab	< 0.0001	13.3
Flavour																
Flavour intensity	65.1abc	35.3g	51.8e	52.3e	47.7ef	51ef	63.8bcd	75.3a	40.9fg	56.5cde	71.7ab	54.5de	63.9bcd	67.5ab	< 0.0001	10.6
Sour fruit	48.3bcdef	57.3abc	66.7a	54.8abcd	63.4ab	67a	40.2defg	28g	53.5abcde	37.8efg	36.5fg	51.3abcdef	45.8cdef	29g	< 0.0001	15.7
Dried fruit	33.8cde	20.1e	25.1de	31.6cde	21.5e	19.6e	45.3abc	58.5a	30.4cde	45abc	50.9ab	25.9de	38.8bcd	54.8a	< 0.0001	15.5
Red fruit	46.0	35.5	39.5	43.5	25.3	53.1	32.4	34.1	30.0	38.2	39.0	39.5	40.5	37.3	0.1105	15.4
Dark fruit	52.1abcde	35.8ef	39.7def	48.5bcdef	41def	33f	63.6ab	68.5a	45.9cdef	53.3abcd	60.8abc	40.9def	55.4abcd	66a	0.0001	16.6
Confectionery	47.4ab	16.8fg	26.3defg	29.7cdef	15.8g	32.6cde	40.4abc	49.8a	20.5efg	33.8cde	47.5a	34.2cde	34bcd	50.8a	< 0.0001	13.4
Mixed spice	36.6bc	18.8e	21.5de	32.8cd	23.5de	21.9de	36.1bc	50.4a	22.4de	33cd	42abc	31.6cd	38.4abc	46.2ab	< 0.0001	12.4
Savoury	28.9	32.7	35.7	27.3	33.4	29.7	42.1	34.6	30.3	33.6	30.1	25.7	30.7	35.7	0.7806	14.2
Sweaty	25.6bc	38ab	37.9ab	34.4ab	47.1a	31.4bc	35.1ab	29.9bc	36.2ab	31bc	19.6c	28.5bc	25.6bc	27.3bc	0.0477	14.1
Green	29.8bcde	44.9a	42.5ab	39ab	48.3a	38.3abc	23.3de	19.2e	34.5abcd	23.1de	18.6e	30.4bcde	23de	24.2cde	0.0001	14.2
Alcohol	62.8bc	23.8gh	35.3fg	47.5def	26.6gh	41.6ef	55.3cd	83a	19.8h	48.6de	74.5ab	36.3efg	57.3cd	72.9ab	< 0.0001	12.6
Mouthfeel																
Acidity	62.5	50.3	59.3	66.7	61.6	57.4	54.8	55.2	46.5	48.8	54.1	38.0	51	50.7	0.1387	17.0
Body	53.3cd	20.4f	29.3ef	37.3e	25.3f	37e	50.8d	74.7a	21.8f	38.8e	63.3bc	38.3e	50d	64.3ab	< 0.0001	10.8
Astringency	53.5bc	22g	38.3ef	43cde	30.5fg	40.9def	52.8bc	66.5a	20g	44.1cde	60.9ab	35.8ef	50.1bcd	60.6ab	< 0.0001	11.3

[1] Values are means of 3 replicates. Different lower case letters within a row indicate significantly different means ($p \le 0.05$, one way ANOVA, post hoc Fisher's LSD).
